# Acute Budd–Chiari syndrome due to a simple liver cyst

**DOI:** 10.1308/003588414X13824511649698

**Published:** 2014-01

**Authors:** J Long, H Vaughan-Williams, J Moorhouse, H Sethi, N Kumar

**Affiliations:** Cardiff and Vale University Health Board,UK

**Keywords:** Budd–Chiari syndrome, Hepatic cyst, Hepatic outflow obstruction

## Abstract

Simple liver cysts are common, rarely causing significant morbidity or mortality. Budd–Chiari syndrome (BCS) is caused by obstruction of hepatic venous outflow and is the leading cause of postsinusoidal liver failure. We present a rare case of BCS caused by a simple hepatic cyst.

A 16cm × 16cm liver cyst was found on computed tomography of a 66-year-old woman presenting with abdominal pain. The cyst had become infected, thus enlarged, exerting mass effect with almost complete compression of the inferior vena cava. Shortly after admission, the patient developed acute liver failure, with deranged clotting and hepatic encephalopathy requiring full organ support on the intensive care unit. Cardiac output studies showed a low cardiac index of 1.4l/min/m^2^.

An emergency laparotomy with fenestration of the cyst and drainage of 2l of purulent material led to a full recovery. Intraoperative cystic fluid aspirates later confirmed no evidence of *Echinococcus*. Histology confirmed a simple cyst. Liver biopsies showed severe, confluent, bridging necrosis, without background parenchymal liver disease.

Acute BCS due to rapid compression of all major hepatic veins leading to fulminant hepatic failure is rare. Our case highlights a clinically significant complication of a simple liver cyst of which clinicians should be aware when managing these ‘innocent’ lesions.

Simple liver cysts are common and estimated to occur in 0.1–2.5% of the population.^1^ The vast majority are asymptomatic and do not require surgical intervention. Budd–Chiari syndrome (BCS), caused by obstruction of hepatic venous outflow, is the leading cause of postsinusoidal liver failure.^2^ Although more commonly caused by intraluminal obstruction such as thrombus, it can be caused by extraluminal compression. We report an isolated case of a patient with this complication from a simple hepatic cyst.

## Case history

**Figure 1:**
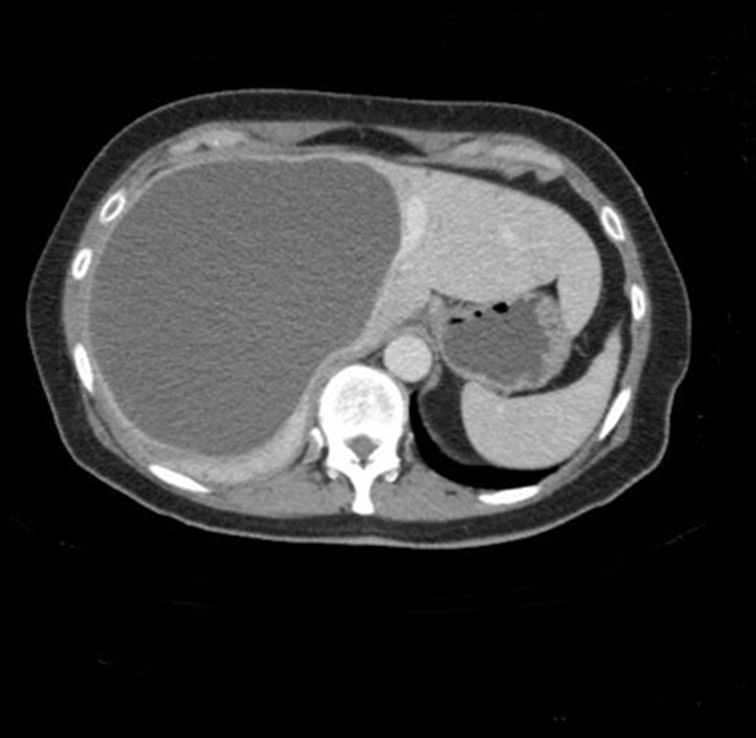

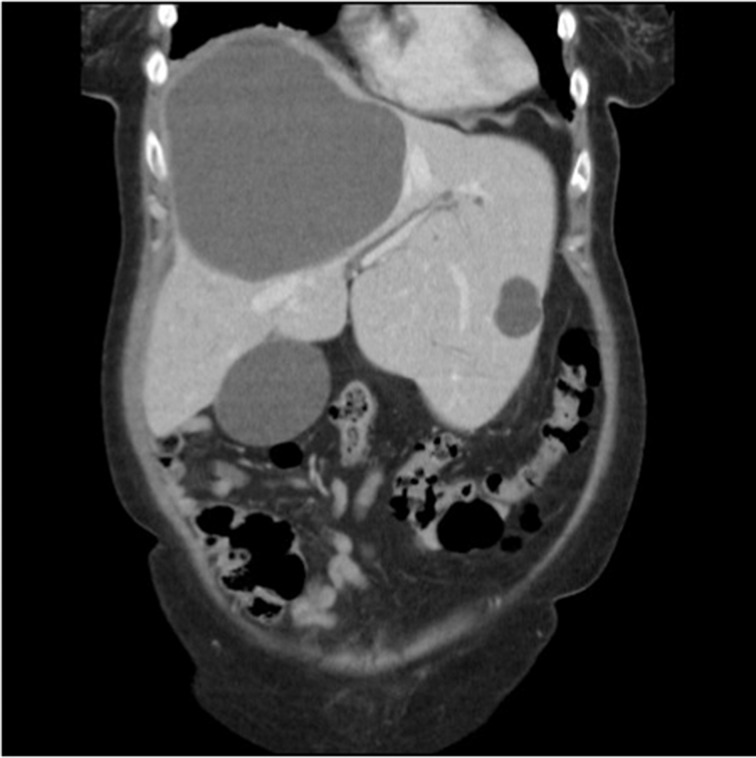
Contrast enhanced computed tomography transverse (left) and coronal (right) section showing 16cm × 16cm cystic lesion in right lobe of liver

A 66-year-old woman was referred to a tertiary liver centre with a short history of nausea and right upper quadrant pain radiating to the back. A large epigastric mass could be felt on examination. Serum inflammatory markers were raised (white cell count of 19.2 × 10^9^/l and C-reactive protein of 210mg/l); other blood tests were normal. Contrast enhanced computed tomography showed a 16cm × 16cm cystic lesion in the right lobe of the liver, exerting significant mass effect with almost complete compression of the inferior vena cava and portal vein bifurcation (Fig 1). A hydatid cyst could not be ruled out on radiology. Serology was therefore awaited prior to attempting percutaneous aspiration.

One day after admission to our unit,the patient became profoundly confused. This was attributed initially to new hyponatraemia of unknown cause (sodium 112mmol/l) and sepsis secondary to the infected cyst. Worsening liver function with elevated enzymes and deranged clotting (alanine transaminase [ALT] 2,650iu/l and prothrombin time 35.7seconds) supported a diagnosis of acute liver failure with hepatic encephalopathy.

The patient developed multiorgan dysfunction requiring inotropic, ventilatory and renal support on the intensive care unit. Cardiac output studies showed a cardiac index of 1.4l/min/m^2^ (normal range: 2.5–4.0l/min/m^2^).

Bedside ultrasonography confirmed the large cystic structure with increased echogenicity. No hepatic vein flow could be identified, in keeping with hepatic outflow obstruction (secondary BCS).

The patient became increasingly unstable and hypotensive, and poor ventilation resulted in an emergency laparotomy, performed via a rooftop incision. Two litres of foul smelling, purulent material was drained from the cyst, the cyst wall was excised and the abdominal cavity was washed out thoroughly. Hydatid precautions, including scolicidal agents, were used in theatre. Liver biopsies were taken to look for underlying liver disease. Postoperatively, the patient returned to the intensive care unit for continued organ support; her cardiac index rapidly returned to normal (4.0l/min/m^2^). She subsequently made a slow but full recovery.

### Pathological findings

**Figure 2:**
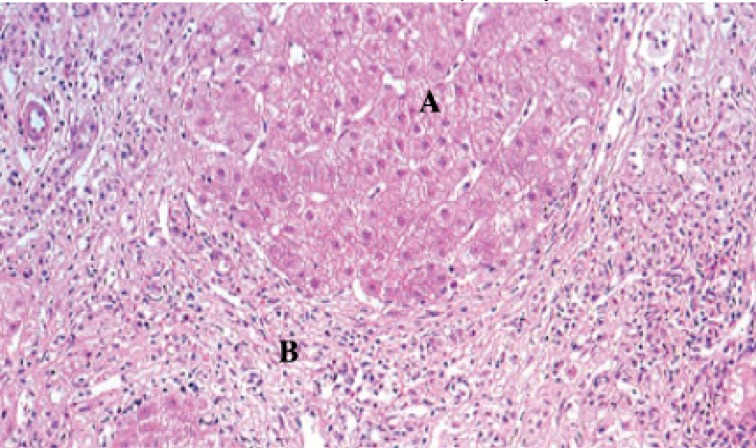
Photomicrography (100x magnification) of biopsy of background liver taken intraoperatively demonstrating severe, confluent, bridging necrosis in a predominantly centrilobular distribution, with no evidence of parenchymal liver disease

Cystic fluid aspirates taken intraoperatively later confirmed no evidence of *Echinococcus* on parasitology studies and cultured mixed coliforms. The fibrous cyst wall contained inflammatory cells and foci of hepatocytes with no evidence of malignancy, confirming a simple liver cyst. Liver biopsies showed severe, confluent, bridging necrosis in a predominantly centrilobular distribution, with no evidence of parenchymal liver disease (Fig 2).

## Discussion

Cystic lesions of the liver differ widely in presentation, aetiology and management. The majority are incidental findings, rarely associated with significant morbidity or mortality and do not warrant surgical intervention.^3^ Simple hepatic cysts are saccular fluid filled structures that do not have septations, calcification, split walls or communicate with the biliary tree. Simple cysts are common, estimated to occur in 0.1–2.5% of the population^1^ based on autopsy and ultrasonography studies, and are more prevalent in women; few are large lesions capable of causing symptoms such as abdominal pain or nausea. Diagnosis of a simple cyst can prove challenging with differential diagnoses of hydatid disease (echinococcal parasite), cystadenoma, cystadenocarcinoma, hepatic abscess, necrotic tumour or other rare malignant cystic lesions. Diagnosis is confirmed with ultrasonography and magnetic resonance imaging.

A simple cyst is a well circumscribed, water attenuated lesion that does not enhance with intravenous contrast. The majority of simple hepatic cysts require no surveillance imaging. However, ambiguity surrounding the diagnosis warrants an interval scan to look for change in size and characteristics of the cyst. Percutaneous needle aspiration of simple cysts with or without injection of sclerosing agents is associated with high recurrence rates. Consequently, first-line management for symptomatic cysts is usually fenestration, commonly via a laparoscopic approach, suitable for over 94% of cases, with low complication rates.^3^ Complications of simple cysts are rare and include infection, spontaneous haemorrhage, rupture into peritoneal cavity or biliary tree, or external compression of biliary tree or major vessels.^4^ These complications are commonly due to anatomical location of the cyst in the hilar region.

In 1845 George Budd described the triad of ascites, abdominal pain and hepatomegaly, and in 1899 Hans Chiari reported the histopathological features of the subsequently named Budd–Chiari syndrome.^5^ BCS is ischaemic parenchymal necrosis followed by hepatic fibrosis and it is one of many causes of acute liver failure. It is caused by obstruction of the hepatic venous outflow at any level from the small hepatic veins to the junction of the inferior vena cava with the right atrium. This can be primary due to intraluminal thrombosis or, more rarely, secondary due to external compression from an abscess, solid tumour or cyst.^2^ It usually takes an insidious course owing to occlusion of small hepatic veins with or without portal vein thrombosis.

The acute form is uncommon, and patients can rapidly develop acute liver failure with features of hepatic encephalopathy, renal failure and coagulopathy with markedly raised ALT levels.^5^ In our case, the hepatic cyst was probably longstanding and only became symptomatic after it was infected. It therefore began to enlarge rapidly. As a consequence, the hepatic venous system became obstructed, causing a fulminant BCS^2^ with hepatocellular ischaemia and associated rapid rise in serum ALT, deranged clotting, acute encephalopathy and fall in cardiac output; cardiac index subsequently improved almost instantly with removal of the obstructing cyst.

## Conclusions

Rapid occlusion of all major hepatic veins leading to fulminant hepatic failure is rare.^2^ We could find no other such published case caused by a simple liver cyst in the English literature. Our case highlights that a benign simple cyst, if large enough or in certain anatomical locations, can cause significant morbidity and clinicians should be aware of this when managing these ‘innocent’ lesions.
